# Elucidating the role of angiogenesis-related genes in colorectal cancer: a multi-omics analysis

**DOI:** 10.3389/fonc.2024.1413273

**Published:** 2024-06-19

**Authors:** Hao-tang Wei, Li-ye Xie, Yong-gang Liu, Ya Deng, Feng Chen, Feng Lv, Li-ping Tang, Bang-li Hu

**Affiliations:** ^1^ Department of Gastrointestinal Surgery, Third Affiliated Hospital of Guangxi Medical University, Nanning, China; ^2^ Department of Research, Guangxi Medical University Cancer Hospital, Nanning, China; ^3^ Department of Information, Library of Guangxi Medical University, Nanning, China

**Keywords:** colorectal cancer, angiogenesis, machine learning algorithms, multi-omics analysis, signature

## Abstract

**Background:**

Angiogenesis plays a pivotal role in colorectal cancer (CRC), yet its underlying mechanisms demand further exploration. This study aimed to elucidate the significance of angiogenesis-related genes (ARGs) in CRC through comprehensive multi-omics analysis.

**Methods:**

CRC patients were categorized according to ARGs expression to form angiogenesis-related clusters (ARCs). We investigated the correlation between ARCs and patient survival, clinical features, consensus molecular subtypes (CMS), cancer stem cell (CSC) index, tumor microenvironment (TME), gene mutations, and response to immunotherapy. Utilizing three machine learning algorithms (LASSO, Xgboost, and Decision Tree), we screen key ARGs associated with ARCs, further validated in independent cohorts. A prognostic signature based on key ARGs was developed and analyzed at the scRNA-seq level. Validation of gene expression in external cohorts, clinical tissues, and blood samples was conducted via RT-PCR assay.

**Results:**

Two distinct ARC subtypes were identified and were significantly associated with patient survival, clinical features, CMS, CSC index, and TME, but not with gene mutations. Four genes (S100A4, COL3A1, TIMP1, and APP) were identified as key ARCs, capable of distinguishing ARC subtypes. The prognostic signature based on these genes effectively stratified patients into high- or low-risk categories. scRNA-seq analysis showed that these genes were predominantly expressed in immune cells rather than in cancer cells. Validation in two external cohorts and through clinical samples confirmed significant expression differences between CRC and controls.

**Conclusion:**

This study identified two ARG subtypes in CRC and highlighted four key genes associated with these subtypes, offering new insights into personalized CRC treatment strategies.

## Introduction

Colorectal cancer (CRC) ranks as a major cause of cancer-related morbidity and mortality worldwide. It displays a wide range of molecular heterogeneity, underscoring the complexity of its pathobiology (1). Despite the progress in genomic profiling, there remain significant challenges in accurately predicting outcomes and tailoring specific therapies for individual patients. Numerous factors contribute to the development, progression, and prognosis of CRC. Among these, angiogenesis is a pivotal process in tumor growth and spread, enabling cancer cells to access the bloodstream and metastasize (2). Angiogenesis, the formation of new blood vessels from existing vasculature, is crucial in both healthy and diseased states (3, 4). It has been firmly established that angiogenesis underpins tumor expansion and dissemination by supplying essential nutrients and oxygen, affirming its status as a fundamental characteristic of cancer and other diseases (5).

Numerous genes and signaling pathways have been identified as critical regulators of angiogenesis, many of which are frequently overexpressed within tumors, thereby amplifying angiogenic signaling (6). In the context of CRC, several genes and molecular pathways such as vascular endothelial growth factor (VEGF) (7), platelet-derived growth factor (PDGF) (8), and fibroblast growth factor (FGF) (9), have been reported as associated with angiogenesis. As a result, targeting angiogenesis has emerged as a promising therapeutic strategy in CRC treatment. For instance, anti-angiogenic drugs like bevacizumab, a monoclonal antibody against VEGF, have demonstrated enhanced patient outcomes when used in conjunction with traditional chemotherapy regimens (10, 11).

Molecular subtyping of CRC has been a significant focus in recent research, greatly enhancing our understanding and management of the disease. CRC is composed of several molecularly distinct subtypes, each characterized by specific genetic and epigenetic alterations. These subtypes have demonstrated substantial influence over prognosis, response to therapy, and overall clinical outcomes (12, 13). Recent studies have leveraged gene profile datasets to develop angiogenesis-related molecular subtypes, with the aim of investigating associations with CRC’s clinical characteristics, prognosis, and tumor microenvironment (TME) (14, 15). However, there still remain numerous angiogenesis genes related to CRC development that require further elucidation. Moreover, the role of angiogenesis within CRC necessitates additional validation through independent cohorts and clinical samples. In light of these gaps in knowledge, we undertook an analysis of angiogenesis-related gene subtypes within CRC. Our primary objective was to ascertain the role of the angiogenesis subtype in the treatment of CRC and identify potential targets for future therapeutic interventions.

## Materials and methods

### Bulk RNA expression acquisition

The RNA-seq data of TCGA-COADREAD dataset and the corresponding clinical features data were achieved from UCSC Xena (https://xenabrowser.net/), which included the data of 576 CRC patients. Three GEO datasets, including GSE41258 (n=390), GSE152430 (n=49), and GSE17538 (n=244) with the survival data of CRC patients were collected from GEO database (https://www.ncbi.nlm.nih.gov/geo/). Data of GSE164191 with blood samples of CRC and control were also collected. In addition, we downloaded two gene expression datasets, namely, IMVigor210 and GSE78220, comprising the data of cancer patients who underwent immunotherapy. These data were utilized to elucidate the role of ARC in the context of immune response modulation. In total, we obtained the characteristic of 1,779 subjects, the baseline of which is shown in **Supplementary Table S1**. The GEO datasets were preprocessed by performing background adjustment using the RMA algorithm. The 36 angiogenesis-related genes (HALLMARK_ANGIOGENESIS) were acquired from the MSigDB database.

### Consensus clustering analysis

Consensus clustering of the CRC dataset samples was using the k-means method with the “ConsensusClusterPlus” package to classify angiogenesis-related clusters (ARC) with CRC, the parameters of the methods were set as default. The optimal number of the clustering was determined by the consensus heatmap and cumulative distribution function (CDF) curves, which was indexed by k values from 2 to 9.

### Gene Ontology of angiogenesis-related genes

GO functional enrichment analysis of biological process of angiogenesis-related genes were performed by the “clusterProfiler” and “org.Hs.eg.db” packages. Significant terms were identified with a cutoff p-value of <0.01 and a false discovery rate of <0.05.

### Calculation of cancer stem cell index, CMS, and gene mutation

Cancer stem cell (CSC) index was represented by stemness indices mRNA stemness index (mRNAsi) and DNA methylation-based stemness index (mDNAsi), which train a stemness signature using normal stem cells and apply the one-class algorithm to define a stemness index for each tumor sample. “CMScaller” package was used to identify CRC patient’s consensus molecular subtypes (CMS) attribution in TCGA-COADREAD dataset, including (CMS1 to CMS4). The somatic mutation data and tumor mutation burden (TMB) data of CRC were collected from “maftools” package, which was from the GDC TCGA database. The mutation status of each cluster was drawn by waterfall plots to demonstrate the somatic mutation of CRC patients in TCGA database.

### Tumor microenvironment estimation

Two algorithms were used to inference TME of CRC by analyzing the TCGA-COADREAD dataset. The Cibersort algorithm was employed to calculate the immune infiltration cells faction in the tissues, which contains 22 types of immune cells. The tumor immune scores were evaluated by ESTIMATE algorithm; the results included stromal score, immune score, ESTIMATE score, and tumor purity.

### Screening important genes of ARC by machine learning methods

Three machine learning algorithms, including least absolute shrinkage and selection operator (LASSO) regression, random forest (Boruta), extreme gradient boosting (XGBoost), and Decision Tree (DT) machine learning algorithms, were applied to screen key genes associated the ARC. The machine learning algorithm steps included loading and cleaning the dataset, dividing the dataset into training and testing sets at a ratio of 7:3, and finally tuning and running the model. The parameters of each step were set as default. We then used overlapping genes from these three algorithms to build a predictive model for the ARC using logistic regression analysis, and estimated the predictive value of the common genes using receiver operating characteristic (ROC) curves and the area under the curve (AUC) measurements.

### Construction of ARC signature for the prognosis of CRC patients

The ARC signature for the survival of CRC patients was also constructed by the common genes from the three machine learning methods. The multivariate Cox regression model was used to construct ARC signature incorporated in the common genes. The prognostic value of ARC signature was estimated by AUC of ROC.

### Analysis CRC scRNA-seq dataset

The scRNA-seq dataset of GSE132465 and annotation data were downloaded from GEO database, which included data of 23 primary colorectal cancer and 10 matched normal mucosa samples. The Seurat 4.2.0 R package was carried out to the processing of the scRNA-seq data. In brief, we performed quality control to filter out low-quality cells. This is followed by data normalization to mitigate the influence of technical variations. “Harmony” function was used to integrate the scRNA-seq data from different samples. Next, we identified highly variable genes to focus on those most likely to be informative for clustering. Principal component analysis (PCA) was then applied to reduce the dataset into a more manageable size while retaining most of the variability. Finally, the PCA result was visualized through UMAP algorithms. The markers used for cell identity were obtained from the ScType package (16).

### Immuno- and chemotherapeutic response prediction

To explore the potential immunotherapy response, we introduce IMvigor210 cohort, which included data from patients with different types of cancer (bladder, kidney, liver, lung lymph node, and ureter) who underwent immune treatment, and GSE78220, which included 28 melanoma patients who underwent PD-1 treatment. The association of ARC with the survival of patients and treatment response was analyzed. In addition, to excavate chemotherapeutic drugs sensitivity to ARC, we computed the semi-inhibitory concentration (IC50) values of common medicines using the “oncoPredict” package. The “oncoPredict” package is an R package for predicting *in vivo* or cancer patient drug response and biomarkers from cell line screening data, and it has been widely applied to various *in vitro* and *in vivo* contexts for drug and biomarker discovery (17).

### Clinical tissue and blood samples collection

A total of 60 CRC tissues and corresponding adjacent tissues were collected from January 2021 to February 2022 in the hospital. CRC patients included in the study had not undergone any prior treatment before surgery and were excluded if they had a history of immune disorders, inflammatory diseases, or severe dysfunction of major organs. Fresh tissues were frozen immediately after surgery and stored in liquid nitrogen until used. In addition, blood samples of 60 CRC patients and 60 non-cancer patients were collected at the same period. The non-cancer patients included patients with hypertension, diabetes, and gastritis. A 10-mL peripheral venous blood sample was collected with EDTA anticoagulant tube and applied for the peripheral blood mononuclear cell (PBMC) separation. PBMC was stored at the temperature of −80℃ until further processing. This study was approved by the hospital’s ethics committee, and written informed consent was obtained from each patient. The clinical features of clinical tissues and plasma samples are listed in **Supplementary Tables S2**, **S3**.

### Reverse transcription-polymerase chain reaction assay

Expression of genes tested by reverse transcription-polymerase chain reaction (RT-PCR). Total RNA from the tissues and the PBMC was isolated using TRIzol reagent (Thermo Fisher Scientific, USA) according to the manufacturer’s instructions. The primers of genes used for RT-PCR are listed in **Supplementary Table S4**. RT-PCR was performed using the SYBR ® Premix Ex Taq kit (Takara, Dalian, China). The relative expression of each gene was calculated using the 2^−ΔΔCT^ method.

### Statistical analysis

Wilcoxon rank-sum test was applied to show the difference between the two groups. Kruskal–Wallis H-test was performed to compare three or more groups. Dunn test was used for multiple comparisons. Chi-square test was used in the comparison of observed and expected results. Kaplan–Meier curves were drawn to exhibit the survival of each group, and log-rank test method was employed to compare survival data. All of the statistical analyses were conducted using R 4.1.3 (p< 0.05).

## Results

### Classification of angiogenesis-associated molecular clusters in CRC

**Figure 1:**
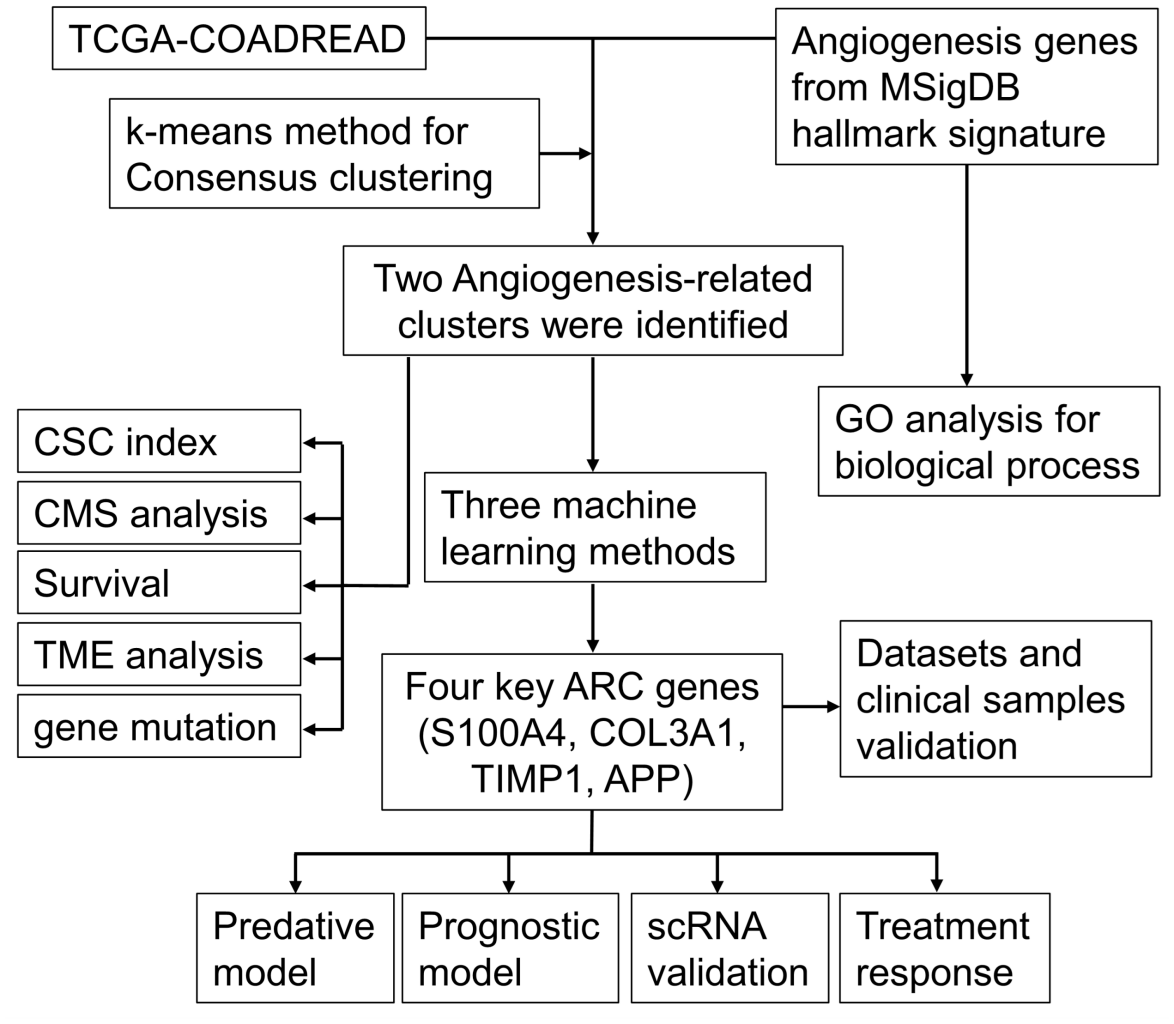
The workflow chart of the study. CSC, cancer stem cell; CMS, consensus molecular subtypes; ARC, angiogenesis-related clusters.

**Figure 2:**
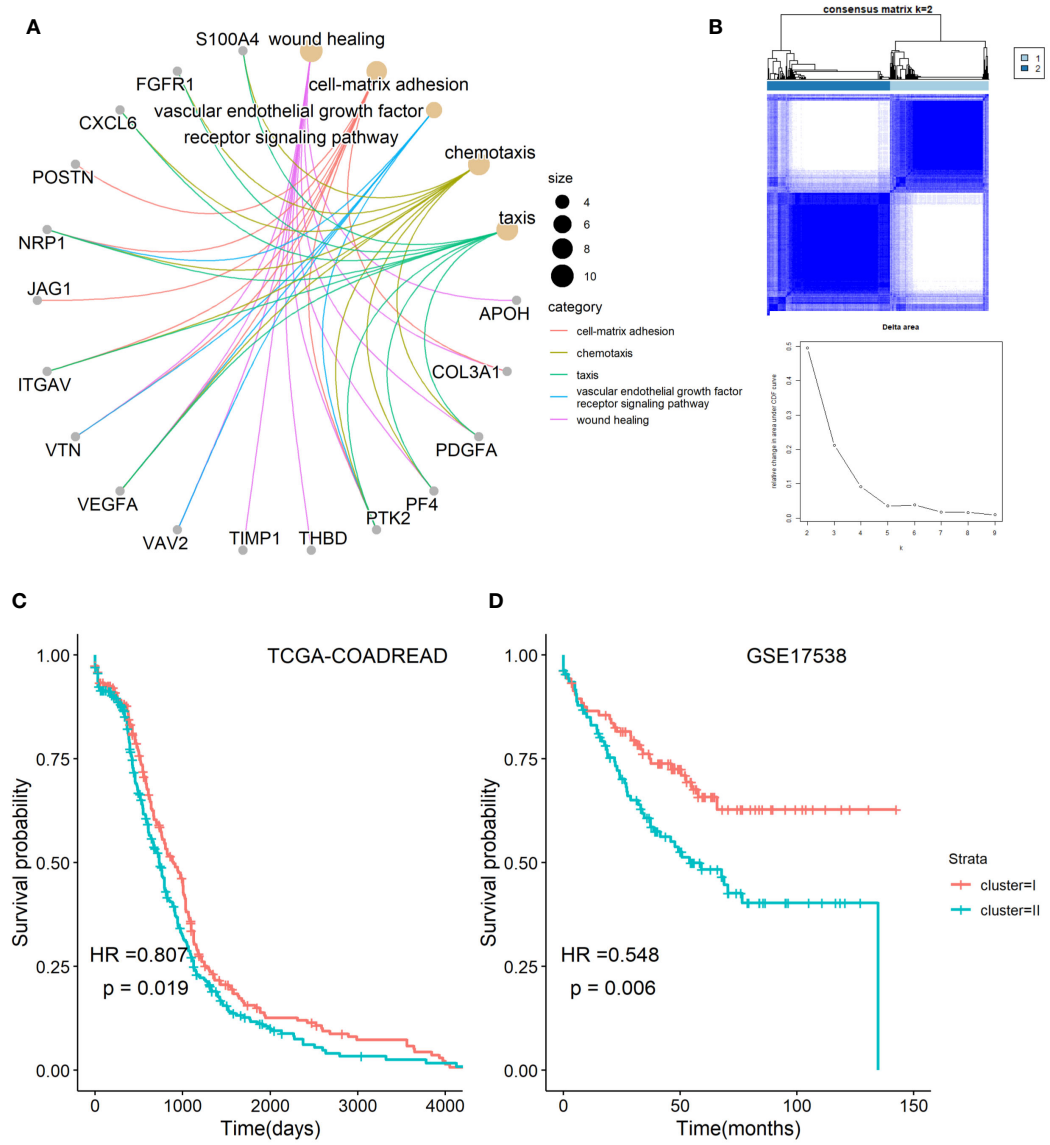
Classification of angiogenesis-related genes clusters in CRC. **(A)** Biological process of angiogenesis-related genes; **(B)** two classification of angiogenesis-related genes were identified by consensus clustering method; **(C,D)** comparison of survival time between cluster I and cluster II of angiogenesis-associated clusters in TCGA-COADARED dataset and GSE17538 dataset.

The workflow chart of the study is shown in **Figure 1**. Employing GO enrichment analysis, we found that the 36 ARGs were mostly enriched in wound healing, cell-matrix adhesion, vascular endothelial growth factor receptor signaling pathway, chemotaxis, and taxis (**Figure 2A**). To investigate the role of ARGs in oncogenesis, we used consensus clustering method to categorize CRC patients from TCGA-COADREAD dataset. This dataset included data from a large-scale of CRC patients, which enhanced the robustness of our results compared to studies with smaller sample sizes. Two angiogenesis-associated cluster (ARC) subtypes were identified (cluster I and cluster II; **Figure 2B**). To explore the prognosis of ARC, Cox analysis and KM plot were employed, and the results showed that patients at cluster I present better survival time than those at cluster II regarding the OS of CRC patients (**Figure 2C**). We also clustered GSE17538 dataset using the same method and found that the OS of CRC patients was significantly poor in cluster II compared with that in cluster I (**Figure 2D**). Collectively, these results indicated that ARC subtypes were able to predict the survival of CRC patients.

### Association of the ARC with clinical parameters and CSC index, CMS in CRC

**Figure 3:**
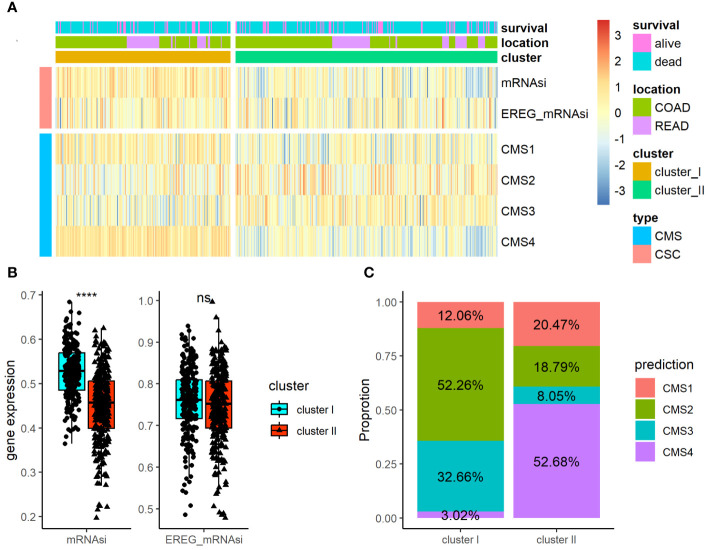
Association of the ARC with clinical parameters and CSC index and CMS in CRC. **(A)** A heatmap characterizing the CSC index and CMS of the ARC; **(B)** comparison of CSC index (miRNAsi and EREG_mRNAsi) between cluster I and cluster II; **(C)** comparison of the proportion of CMS between cluster I and cluster II. CSC, cancer stem cell; CMS, consensus molecular subtypes. ****p<0.00001, ns, not significance.

Using the TCGA-COADREAD dataset, we determined the association of the ARC with clinical parameters in CRC patients. Besides the common clinical parameters, CSC index has been considered promising therapeutic targets for cancer therapy. CMS classification system is currently available for CRC with clear biological interpretability and subtype-based targeted interventions. As listed in **Supplementary Table S5**, there were significant differences between cluster I and cluster II regarding T stage and N stage of CRC, but no greatly difference regarding patients’ age, gender, location, histological type, M stage, and tumor stage. Furthermore, we examined the associations of ARC with CMS and CSC index (included mRNAsi and EREG mRNAsi index) in CRC patients (**Figure 3A**); the results showed that cluster I showed higher mRNAsi index than cluster II, but there was no significant difference regarding EREG mRNAsi index between cluster I and cluster II (**Figure 3B**). In addition, there were significant differences between cluster I and cluster II regarding CMS proportion of CRC, and cluster I contained high CMS2 proportion while cluster II contained mostly CMS4 proportion (**Figure 3C**).

### Analysis of ARC with TME and gene mutation in CRC

**Figure 4:**
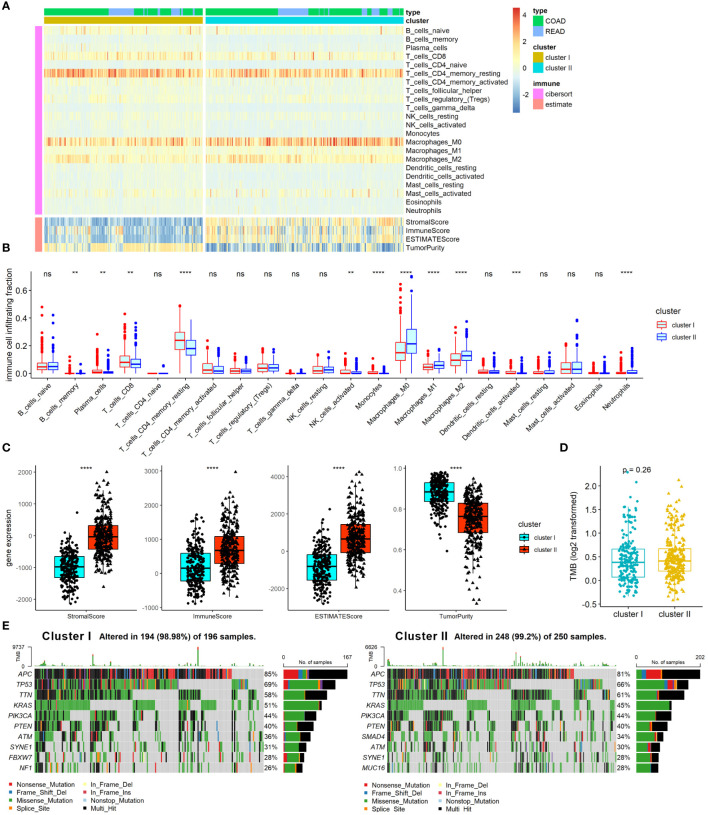
Analysis of ARC with TME and gene mutation in CRC. **(A)** A heatmap characterizing the immune cells and immune score; **(B)** comparison of 22 immune cells proportion between cluster I and cluster II of ARC; **(C)** comparison of tumor immune scores between cluster I and cluster II of ARC; **(D)** comparison of TMB between cluster I and cluster II of ARC; **(E)** gene mutation status of cluster I and cluster II in CRC. TMB, tumor mutation burden. **p<0.001, ***p<0.0001, ****p<0.00001.

TME and gene mutation are known as critical factors that affect CRC pathogenesis, progress, treatment response, and prognosis, and have close connection with angiogenesis in CRC. We therefore further examined the association of ARC with TME and gene mutation in CRC. First, we employed Cibersort algorithm to determine the proportion of 22 immune cells and ESTIMATE algorithm to calculate tumor immune scores in the TME, and the results were presented by heatmap (**Figure 4A**). The results showed that cluster I was significantly enriched in B-cell memory, plasma cells, T cells CD8, T-cell CD4 memory resting, NK cells activated, and monocytes, while cluster II was remarkably enriched in macrophages M0, macrophages M1, macrophages M2, and neutrophils (**Figure 4B**). We also found that tumor immune score of stromal score, immune score, and ESTIMATE score were greatly increased in cluster I compared with those in cluster II, but the tumor purity was high in cluster I compared with that in cluster II (**Figure 4C**). Furthermore, we compared the TMB between cluster I and cluster II, but there was no significant difference between them (**Figure 4D**). Moreover, in line with the results of TMB, there was no considerable difference between cluster I and cluster II regarding the top 10 mutation genes (**Figure 4E**). Collectively, these results indicated that the ARC was associated with the TME, but little associated with gene mutation status of CRC.

### Machine-learning-based establishment of ARC-related signature for CRC

**Figure 5:**
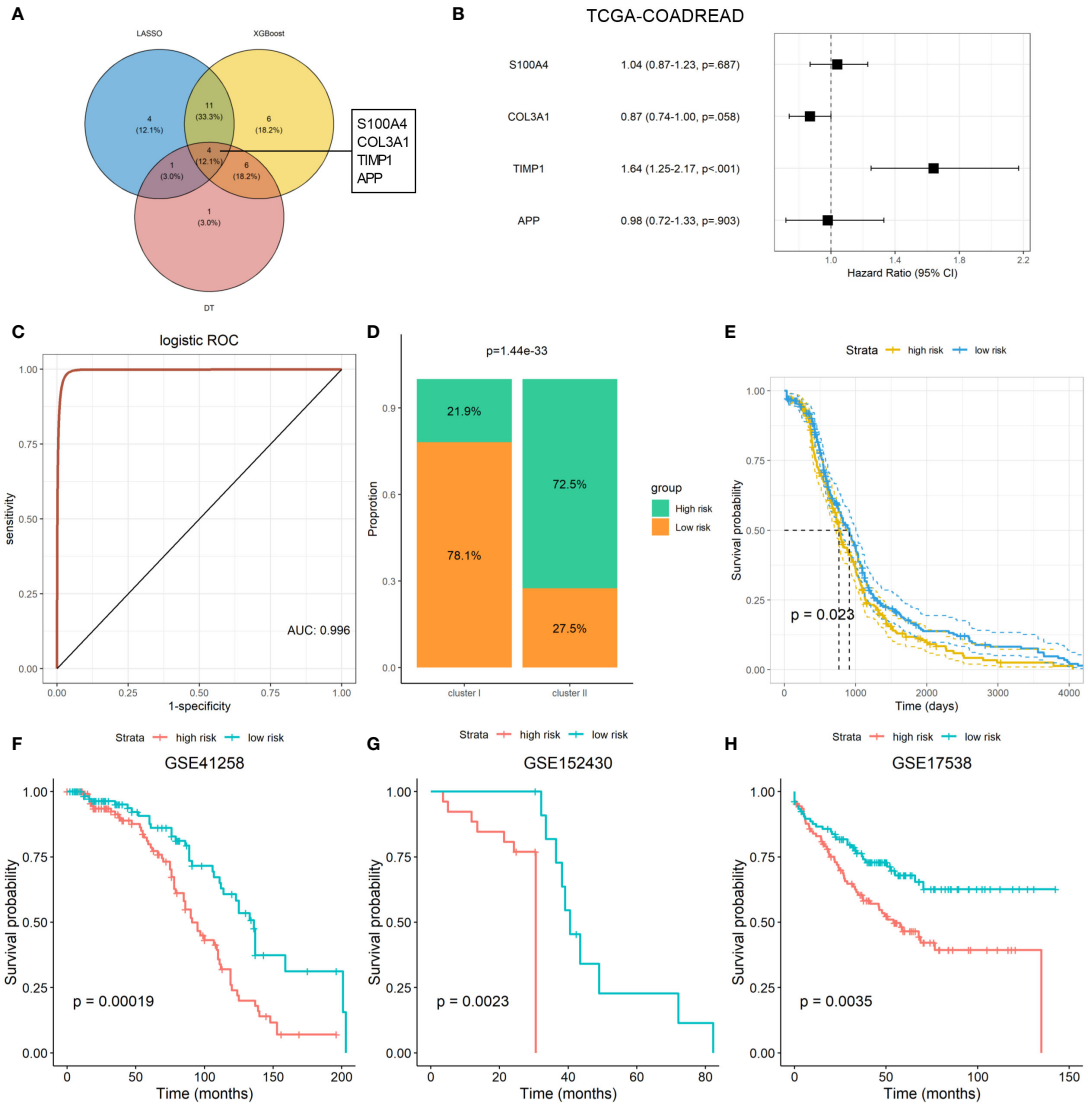
Machine learning-based establishment of ARC prognostic signature for CRC. **(A)** Venn plot of the common genes from important genes of three machine-learning-based algorithms; **(B)** association of four ARC-related genes with the survival of CRC patients; **(C)** predictive value of four ARC-related genes in discriminated ARC subtypes; **(D)** comparison of high- and low-risk proportion between cluster I and cluster II of ARC; **(E)** survival analysis of high- and low-risk of ARC-related signature for CRC in TCGA-COADREAD dataset; **(F–H)** survival analysis of high- and low-risk of ARC prognostic signature for CRC patients in GSE41258, GSE152430, and GSE17538 datasets.

Machine-learning-based gene selection approaches are widely used in current studies, as they have been reported to enhance dimensionality reduction precision (18). Three machine learning algorithms, including LASSO, Xgboost, and DT machine learning algorithms, were applied to explore the important genes of ARGs that were associated with the ARC using TCGA-COADREAD dataset. After overlapping the important genes screening from the three machine learning algorithms, four genes, which included S100 calcium binding protein A4 (S100A4), collagen type III alpha 1 chain (COL3A1), TIMP metallopeptidase inhibitor 1 (TIMP1), and amyloid precursor protein (APP), were identified as the common genes of the three machine learning algorithms (**Figure 5A**). We found that only TIMP1 was significantly associated with the prognosis of CRC patients, while the remaining three genes showed minimal association with their prognosis (**Figure 5B**). Then, logistic regression analysis was performed to determine the predictive value of the four genes in distinguish ARC subtypes. As shown in **Figure 5C**, these four genes could better distinguish cluster I from cluster II, with the AUC value as 0.996.

We next constructed an ARC prognostic signature for CRC using these four genes and divided the CRC patients into high- and low-risk group based on the median value of the signature. We found that patients at high-risk group harbor much more proportion of cluster I than that at the low-risk group (**Figure 5D**) and presented shorter survival time than those at the low-risk group (**Figure 5E**). Finally, we verified the association of the signature with CRC patients’ survival using three independent cohorts, including GSE41258, GSE152430, and GSE17538. By KM plot and log-rank analysis, we found that patients with high risk present shorter survival time than those with low risk (**Figures 5F-H**), suggesting that this signature was able to screen CRC patients at different risk of survival.

### Exploration of four ARC-related gene at scRNA-seq level

**Figure 6:**
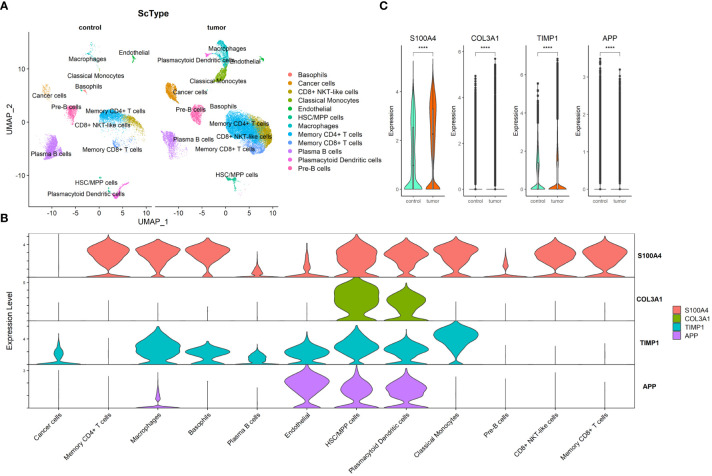
Exploration of four ARC-related genes at scRNA-seq level. **(A)** UMAP visualization of CRC cell clusters between tumor and control tissues at scRNA-seq level. Color codes represent sample types; **(B)** distribution of four ARC genes in 12 cell types; **(C)** comparison of four ARC-related genes in cancer cells between tumor tissues and control tissues. ****p<0.00001.

Through preprocessing of GSE132465 scRNA-seq dataset, 15 cell clusters were identified; then, the cell type annotation was conducted based on “ScType” package and visualized by UMAP plot, and 12 cell types were annotated (**Figure 6A**). As shown in **Figure 6B**, S100A4 was highly expressed in memory CD4+ T cells, macrophages, basophils, HSC/MPP cells, plasmacytoid dendritic cells, classical monocytes, CD8+ NKT-like cells, and memory CD8+ T cells; COL3A1 in HSC/MPP cells and plasmacytoid dendritic cells; TIMP1 in macrophages, basophils, endothelial, HSC/MPP cells, plasmacytoid dendritic cells, and classical monocytes; and APP in endothelial, HSC/MPP, and plasmacytoid dendritic cells. We compared the expression of the four ARC-related genes between cancer and control tissues in cancer cells and observed that expression of S100A4, COL3A1, and TIMP1 in cancer cells was significantly elevated in cancer tissues compared with that in control tissues, but the expression of APP was lower in in cancer tissues than in control tissues (**Figure 6C**).

### Determination of ARC with the survival and immunotherapy response

**Figure 7:**
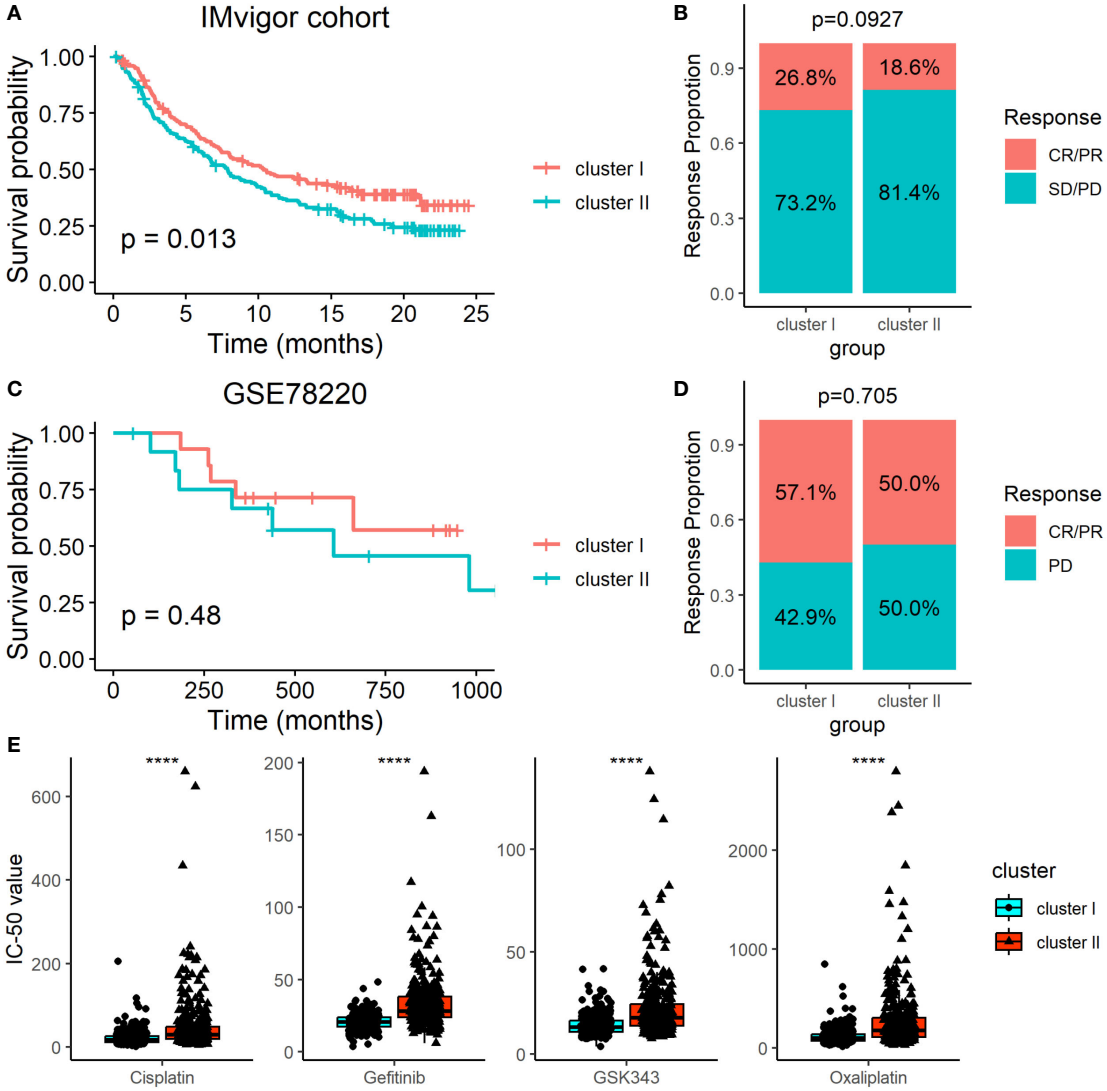
Determination of ARC with the survival and immunotherapy response. **(A)** Survival analysis of different ARC with the immunotherapy in IMvigor cohort; **(B)** comparison of immunotherapy response between cluster I and cluster II in IMvigor cohort; **(C)** survival analysis of different ARC with the immunotherapy in GSE78220 dataset; **(D)** comparison of immunotherapy response between cluster I and cluster II in GSE78220 dataset; **(E)** comparison of IC50 of four chemotherapeutic medicines between cluster I and cluster II. CR, complete response; PR, partial response; SD, stable disease; PD, progression disease; ****p<0.00001.

Since the ARC was associated with the TME, we therefore determined the association of the ARC subtypes with immunotherapy by analyzing the data from the IMvigor cohort, which included multiple cancer types in patients who underwent immunotherapy. Although patients’ survival of cluster I was longer than that of cluster II (**Figure 7A**), we failed to find that there was significant treatment response between cluster I and cluster II (**Figure 7B**). We also employed another dataset, GSE78220, which included 28 melanoma patients who underwent PD-1 treatment, but neither the survival time nor the treatment response show significant difference between cluster I and cluster II (**Figures 7C, D**). Additionally, we explored the potential treatment for each ARC by analyzing the sensitivity of typical chemotherapeutic medicines. The “oncoPredict” package was used to implement the treatment prediction for each ARC showing drug sensitivity in the form of IC50. The results showed that cisplatin, gefitinib, GSK343, and oxaliplatin present much lower IC50 value in cluster I than in cluster II (**Figure 7E**), suggesting that patients at cluster I might be sensitive to these chemotherapeutic medicines.

### Validation of the ARC signature genes datasets and clinical samples

**Figure 8:**
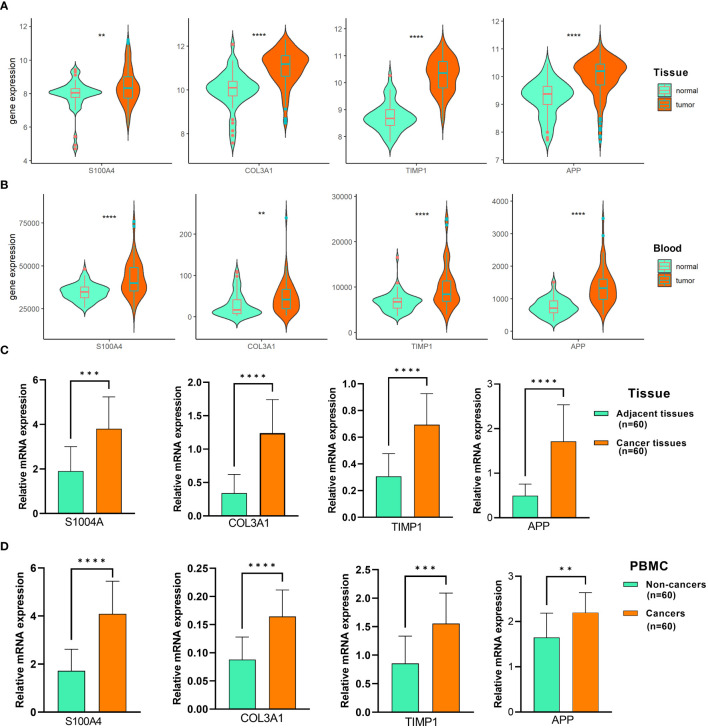
Validation of the ARC signature genes datasets and clinical samples. **(A,B)** Comparison of the expression of S100A4, COL3A1, TIMP1, and APP in **(A)** GSE41258 dataset, **(B)** GSE164191 dataset, **(C)** CRC tissues and adjacent tissues, and **(D)** CRC and non-cancer blood samples. Data were expressed as mean and SD. **p<0.001, ***p<0.0001, ****p<0.00001.

To verify the expression of four ARC signature genes in CRC, we examined their expression in two large samples of external datasets, GSE41258 (181 tumor tissue and 46 control tissue samples) and GSE164191 (59 tumor blood and 62 normal blood samples), and found that S100A4, COL3A1, TIMP1, and APP were all highly expressed in CRC compared with controls between CRC and controls in both tissues and blood samples (**Figures 8A, B**). Next, we examined the expression of the four genes in clinical CRC tissues and blood samples using RT-PCR assay and found that the results were similar to that of CRC datasets (**Figures 8C, D**), with the expression of the four genes significantly increased in CRC compared with the controls, regardless of tumor tissues or blood samples. Taken together, these results suggested that the four ARC signature genes were closely related to the CRC.

## Discussion

Angiogenesis, defined as the formation of new blood vessels from pre-existing vasculature, is a critical component in cancer development and progression. This process facilitates tumor growth and metastasis by supplying rapidly proliferating cancer cells with essential oxygen and nutrients. Analogous to other solid tumors, CRC development, progression, and metastasis are heavily reliant on angiogenesis (19, 20). A plethora of molecules, including growth factors such as epidermal growth factor (EGF), FGF-2, VEGF, transforming growth factor (TGF)-α and TGF-β (21), and angiopoietins (22) participate in this process. These genes orchestrate intricate signaling pathways that promote endothelial cell proliferation, migration, and survival—processes fundamental for angiogenesis. Understanding the molecular mechanisms and genetic regulation underpinning angiogenesis not only sheds light on the pathophysiology across numerous diseases but also opens the possibility for novel therapeutic interventions.

In this study, we segregated CRC patients into two distinct angiogenesis-related subtypes—cluster I and cluster II—using an unsupervised clustering algorithm. Significant disparities were observed between these clusters concerning survival time, mRNAsi, CMS, immune cell fraction, and tumor immune scores. However, minimal differences were noticed regarding EREG-mRNAsi, TMB, and the top 10 gene mutations. These findings align with previous research (14, 15). Utilizing three machine learning algorithms, we identified four genes significantly associated with CRC subtypes. These genes exhibited high predictive value for differentiating the two clusters. Additionally, a signature built using these four genes proved to be effective in stratifying patients into high- or low-risk survival categories, a result corroborated by independent cohorts. Subsequent single-cell RNA-sequencing dataset analysis revealed the primary sources of the four genes. Interestingly, none of these genes showed high expression in cancer cells. Instead, they primarily manifested within various types of immune cells, despite profound expression differences between tumor and control tissues in the cancer cells.

Studies have shown that the presence of tumor-infiltrating lymphocytes, particularly cytotoxic CD8+ T cells, is associated with improved response to immunotherapy across different cancer types (23). Recent therapeutic strategies incorporating immune-checkpoint inhibitors and anti-angiogenic agents have significantly advanced clinical cancer treatment. Numerous studies have explored the interplay between immunity and angiogenesis within the TME, including the relationship of angiogenesis-related genes—such as VEGFA, SPP1, and CXCL16—with immune cells, the TME, and immune therapies (24). For example, CXCL16 facilitates tumor metastasis by modulating angiogenesis within the TME of CRC (25). Members of the VEGF family, induced by hypoxia-inducible factor (HIF) signaling, play a crucial role in angiogenesis by binding to their receptors VEGFR1–2 and neuropilin (26). The interaction between immune cells and CRC cells impacts tumor growth, invasiveness, and angiogenesis (27). Additionally, angiogenesis regulators such as S100A4, SPARC, and SPP1 are associated with macrophage infiltration and serve as prognostic biomarkers in CRC (28). These findings highlight the association of certain angiogenesis-related genes with the TME in CRC.

We also evaluated the immunotherapeutic response of cluster I and cluster II using the IMvigor210 and GSE78220 datasets, as done in previous studies (29, 30). Although we identified an association between the ARC and the TME, our analysis did not reveal a significant correlation between these subtypes and immunotherapy response or patient survival. Given that the patients in these cohorts were not exclusively CRC patients, we hypothesize that the inclusion of patients with other types of cancers might have contributed to the observed discrepancies. Consequently, the precise relationship between ARC subtypes and immunotherapy effectiveness needs to be validated specifically in CRC patients who have received immunotherapy. As our results indicated poorer survival rates in cluster II compared to cluster I, we screened and identified four chemotherapeutic medications sensitive to this cluster, thus providing potential treatment options for these patients. Finally, we examined two larger sample cohorts and confirmed the expression of the four genes in CRC tissues. We also collected clinical CRC tissue and blood samples for further validation of gene expression using RT-PCR assays. This demonstrated the reliability of our results.

Among the four genes (S100A4, COL3A1, TIMP1, and APP) associated with CRC, S100A4 is a notable regulator of angiogenesis. A study reported that S100A4 mediates the effect of STC1 on angiogenesis in breast cancer (31). It also serves as a prognostic biomarker for CRC (28, 32). Furthermore, S100A4 facilitates CRC metastasis via MACC1 (33, 34). COL3A1, encoded by SPARC, is a cysteine-rich acidic matrix-associated protein crucial for extracellular matrix (ECM) remodeling (35). Research has demonstrated that COL3A1 is selectively expressed by the microvasculature in brain tumors (36). Existing evidence indicates that COL3A1 exhibits higher expression in CRC tissues compared to control tissues and is associated with CRC metastasis (37–39). TIMP1, reported to be a multi-faceted biomarker in CRC, can function as a diagnostic biomarker for CRC and is linked to the immunological microenvironment, drug sensitivity, and inhibition of ferroptosis (40). Additionally, TIMP1 is correlated with angiogenesis in CRC (41, 42), gastric cancer (43), and CRC metastasis (44). In addition, TIMP1 was identified to be upregulated in particularly aggressive forms of CRC liver metastases, specifically those with the replacement histopathological growth pattern (45). Amyloid precursor protein (APP), the biological precursor of β-amyloids, has been extensively studied in relation to Alzheimer’s disease (46, 47), where it is involved in angiogenesis (48, 49). However, its role in cancer, particularly CRC, remains vastly unexplored. A recent study revealed that TIMP-1 acts as a novel ligand of APP, triggering a proinflammatory phenotype in human monocytes (50). Moreover, APP mediates amyloid β peptide interaction in basal prostate cancer and mesenchymal colon cancer (51). Collectively, these findings suggest that these four angiogenesis-related genes are significantly involved in the angiogenesis of various diseases, including cancers, and are linked to the pathogenesis of CRC.

While previous studies have reported on the role of angiogenesis-related clusters in CRC (14, 15), these studies lacked clinical samples and larger sample sizes of CRC datasets for gene expression verification. In contrast, our study utilized three machine learning algorithms to screen for important genes, resulting in a smaller but more accurate predictive set compared to previous studies. This makes our findings more readily applicable in clinical practice. Furthermore, our research mapped gene expression at single-cell RNA levels and discovered that these genes mainly manifest in non-cancerous cells, a finding not previously reported. Nonetheless, several limitations in our study should be acknowledged. First, due to a lack of survival data from clinical samples, we were unable to conduct survival analysis for these genes. Second, current data regarding CRC patients who underwent immunotherapy is unavailable. Lastly, while previous studies have reported the role of these angiogenesis-related genes in various diseases, the exact mechanism underlying angiogenesis in CRC needs to be confirmed through *in vivo* and *in vitro* experiments. Therefore, future studies are required to determine the exact association of ARG with treatment response.

## Conclusions

Our study uncovered two ARG subtypes associated with the prognosis and clinical characteristics of CRC. Additionally, we identified potential chemotherapeutic medications for patients. Four key genes related to ARG subtypes were also identified and validated. These findings provide valuable targets for assessing survival outcomes in CRC patients and offer guidance toward individualized treatment strategies.

## Data availability statement

The original contributions presented in the study are included in the article/**Supplementary Material**. Further inquiries can be directed to the corresponding authors.

## Ethics statement

The studies involving humans were approved by Ethics Committee of Guangxi Medical University Cancer Hospital. The studies were conducted in accordance with the local legislation and institutional requirements. The participants provided their written informed consent to participate in this study.

## Author contributions

H-TW: Writing – original draft, Validation, Investigation, Formal analysis. L-YX: Writing – original draft, Validation, Methodology, Formal analysis. Y-GL: Writing – original draft, Methodology, Formal analysis. YD: Writing – original draft, Validation, Methodology. FC: Writing – original draft, Validation, Methodology. FL: Writing – original draft, Validation, Methodology. L-PT: Writing – review & editing, Formal analysis, Conceptualization. B-LH: Writing – review & editing, Funding acquisition, Conceptualization.
