# Supplementary material for: Elucidating the role of angiogenesis-related genes in colorectal cancer: a multi-omics analysis
Source: Front Oncol. 2024 Jun 19;14:1413273. doi: 10.3389/fonc.2024.1413273 (PMC11220232; doi:10.3389/fonc.2024.1413273)
Supplement: Supplementary file 1 [file Table_1.docx]

**Supplement files**

**Table S1** Baseline of the include datasets

| ID | Platform | Samples number | Samples types | Country |
| --- | --- | --- | --- | --- |
| TCGA-COADREDAD | RNA-Seq | 576 | Tissues | USA |
| GSE41258 | GPL96 | 390 | Tissues | Israel |
| GSE152430 | GPL18573 | 49 | Tissues | USA |
| GSE17538 | GPL570 | 244 | Tissues | USA |
| GSE78220 | GPL11154 | 28 | Tissues | USA |
| GSE164191 | GPL570 | 121 | Blood | China |
| IMVigor210 | RNA-Seq | 348 | Tissues | USA |
| GSE132465 | scRNA | 23 | Tissues | South Korea |

**Table S2 Characteristics of the included CRC patients of tissues samples**

| **Variables** | **Values** |
| --- | --- |
| Age (Years) | 63.9 ± 13.5 |
| Gender |  |
| Male | 35 (58.3%) |
| Female | 25 (41.7%) |
| Location |  |
| Colon | 43 (71.7%) |
| Rectal | 17 (28.3%) |
| Grade |  |
| Low | 6 (10.0%) |
| High | 2 (3.3%) |
| Middle | 52 (86.7%) |
| T stage |  |
| T2 | 6 (10.0%) |
| T3 | 26 (43.3%) |
| T4 | 28 (46.7%) |
| N stage |  |
| N0 | 33 (55.0%) |
| N1 | 14 (23.3%) |
| N2 | 13 (21.7%) |
| M stage |  |
| M0 | 48 (80.0%) |
| M1 | 12 (20.0%) |
| Tumor stage |  |
| Ⅰ | 6 (10.0%) |
| Ⅱ | 24 (40.0%) |
| Ⅲ | 18 (30.0%) |
| Ⅳ | 12 (20.0%) |

**Table S2 Characteristics of the included CRC patients of blood samples**

|  | CRC | Non-cancer |
| --- | --- | --- |
| Age (Years) | 55.9 ± 11.6 | 53.2±10.5 |
| Gender |  |  |
| Male | 43 (71.7%) | 40 (66.7%) |
| Female | 17 (28.3%) | 20 (33.7%) |
| Location |  |  |
| Colon | 35 (58.3%) |  |
| Rectal | 25 (41.7% |  |
| T stage |  |  |
| T0 | 2 (3.3%) |  |
| T1 | 1 (1.7%) |  |
| T2 | 3 (5.0%) |  |
| T3 | 29 (48.3%) |  |
| T4 | 25 (41.7%) |  |
| N stage |  |  |
| N0 | 17 (28.3%) |  |
| N1 | 14 (23.3%) |  |
| N2 | 18 (30.0%) |  |
| Nx | 11 (18.3%) |  |
| M stage |  |  |
| M0 | 44 (73.3%) |  |
| M1 | 16 (26.7%) |  |

**Table S4 Sequence of gene primers**

| Gene | Direction | Sequence |
| --- | --- | --- |
| S100A4 | Forward | 5′- CAGGGACAACGAGGTGGACTTC -3′ |
|  | Reverse | 5′- TTTCTTCCTGGGCTGCTTATCTGG -3′ |
| COL3A1 | Forward | 5′- TCCAAAGGGTGACAAGGGTGAAC -3′ |
|  | Reverse | 5′- AGGAGGACCAATAGGACCAGTAGG -3′ |
| TIMP1 | Forward | 5′- CCACAACCGCAGCGAGGAG -3′ |
|  | Reverse | 5′- AACAGTGTAGGTCTTGGTGAAGCC -3′ |
| APP | Forward | 5′- GACGATGAGGATGGTGATGAGGTAG -3′ |
|  | Reverse | 5′- TGGTGGTGGTGGTGGCAATG -3′ |

**Table S5 Association of the ARC clusters with clinical parameters in CRC patients**

| Features | Levels | Cluster I (N=256) | Cluster II (N=320) | P value |
| --- | --- | --- | --- | --- |
| Age | Median (IQR) | 68.0 (59.0 to 75.0) | 67.0 (57.0 to 76.0) | 0.705 |
| Gender | female | 115 (44.9%) | 147 (45.9%) | 0.874 |
|  | male | 141 (55.1%) | 173 (54.1%) |  |
| Cancer type | COAD | 188 (73.4%) | 236 (73.8%) | 1.000 |
|  | READ | 68 (26.6%) | 84 (26.2%) |  |
| Histological type | NA | 4 (1.6%) | 6 (1.9%) | 0.231 |
|  | Adenocarcinoma | 227 (88.7%) | 268 (83.8%) |  |
|  | Mucinous | 25 (9.8%) | 46 (14.4%) |  |
|  | NA | 0 (0%) | 1 (0.3%) | 0.046 |
| T stage | T1 | 10 (3.9%) | 8 (2.5%) |  |
|  | T2 | 54 (21.1%) | 45 (14.1%) |  |
|  | T3 | 172 (67.2%) | 224 (70%) |  |
|  | T4 | 20 (7.8%) | 42 (13.1%) |  |
| N stage | NA | 0 (0%) | 1 (0.3%) | 0.001 |
|  | N0 | 166 (64.8%) | 158 (49.4%) |  |
|  | N1 | 57 (22.3%) | 84 (26.2%) |  |
|  | N2 | 33 (12.9%) | 75 (23.4%) |  |
|  | NX | 0 (0%) | 2 (0.6%) |  |
| M stage | NA | 4 (1.6%) | 3 (0.9%) | 0.720 |
|  | M0 | 191 (74.6%) | 237 (74.1%) |  |
|  | M1 | 32 (12.5%) | 48 (15%) |  |
|  | MX | 29 (11.3%) | 32 (10%) |  |
| Tumor stage | NA | 8 (3.1%) | 12 (3.8%) | 0.092 |
|  | I | 51 (19.9%) | 45 (14.1%) |  |
|  | II | 101 (39.5%) | 109 (34.1%) |  |
|  | III | 64 (25%) | 105 (32.8%) |  |
|  | IV | 32 (12.5%) | 49 (15.3%) |  |

COAD: colon cancer; READ: rectal cancer; NA: not available.
